# Social norms towards smoking and electronic cigarettes among adult smokers in seven European Countries: Findings from the EUREST-PLUS ITC Europe Surveys

**DOI:** 10.18332/tid/104417

**Published:** 2019-03-22

**Authors:** Katherine A. East, Sara C. Hitchman, Mairtin McDermott, Ann McNeill, Aleksandra Herbeć, Yannis Tountas, Nicolas Bécuwe, Tibor Demjén, Marcela Fu, Esteve Fernández, Ute Mons, Antigona C. Trofor, Witold A. Zatoński, Geoffrey T. Fong, Constantine I. Vardavas, on behalf of the EUREST-PLUS consortium

**Affiliations:** 1 National Addiction Centre, Institute of Psychiatry, Psychology and Neuroscience, King’s College London, London, United Kingdom; 2 UK Centre for Tobacco and Alcohol Studies, King’s College London (KCL), London, United Kingdom; 3 Health Promotion Foundation (HPF), Warsaw, Poland; 4 Department of Behavioural Science and Health, University College London, London, United Kingdom; 5 National and Kapodistrian University of Athens (UoA), Athens, Greece; 6 Kantar Public (TNS), Brussels, Belgium; 7 Smoking or Health Hungarian Foundation (SHHF), Budapest, Hungary; 8 Catalan Institute of Oncology (ICO), Barcelona, Spain; 9 Bellvitge Biomedical Research Institute (IDIBELL), Barcelona, Spain; 10 Cancer Prevention Unit and WHO Collaborating Centre for Tobacco Control, German Cancer Research Center (DKFZ), Heidelberg, Germany; 11 University of Medicine and Pharmacy ‘Grigore T. Popa’ Iasi, Iasi, Romania; 12 Aer Pur Romania, Bucharest, Romania; 13 European Observatory of Health Inequalities, President Stanisław Wojciechowski State University of Applied Sciences, Kalisz, Poland; 14 Department of Psychology and School of Public Health and Health Systems, University of Waterloo (UW), Waterloo, Canada; 15 Ontario Institute for Cancer Research, Toronto, Canada; 16 European Network for Smoking and Tobacco Prevention (ENSP), Brussels, Belgium; 17 University of Crete (UoC), Heraklion, Greece

**Keywords:** survey, smoking, Europe, electronic cigarettes, social norms

## Abstract

**INTRODUCTION:**

This study explores whether current smokers’ social norms towards smoking and electronic cigarettes (e-cigarettes) vary across seven European countries alongside smoking and e-cigarette prevalence rates. At the time of surveying, England had the lowest current smoking prevalence and Greece the highest. Hungary, Romania and Spain had the lowest prevalence of any e-cigarette use and England the highest.

**METHODS:**

Respondents were adult (≥18 years) current smokers from the 2016 EUREST-PLUS ITC (Romania, Spain, Hungary, Poland, Greece, Germany) and ITC 4CV England Surveys (N=7779). Using logistic regression, associations between country and (a) smoking norms and (b) e-cigarette norms were assessed, adjusting for age, sex, income, education, smoking status, heaviness of smoking, and e-cigarette status.

**RESULTS:**

Compared with England, smoking norms were higher in all countries: reporting that at least three of five closest friends smoke (19% vs 65–84% [AOR=6.9–24.0; Hungary–Greece]), perceiving that people important to them approve of smoking (8% vs 14–57% [1.9–51.1; Spain–Hungary]), perceiving that the public approves of smoking (5% vs 6–37% [1.7–15.8; Spain–Hungary]), disagreeing that smokers are marginalised (9% vs 16–50% [2.3–12.3; Poland–Greece]) except in Hungary. Compared with England: reporting that at least one of five closest friends uses e-cigarettes was higher in Poland (28% vs 36% [2.7]) but lower in Spain and Romania (28% vs 6–14% [0.3–0.6]), perceiving that the public approves of e-cigarettes was higher in Poland, Hungary and Greece (32% vs 36–40% [1.5–1.6]) but lower in Spain and Romania in unadjusted analyses only (32% vs 24–26%), reporting seeing e-cigarette use in public at least some days was lower in all countries (81% vs 12–55% [0.1–0.4]; Spain–Greece).

**CONCLUSIONS:**

Smokers from England had the least pro-smoking norms. Smokers from Spain had the least pro-e-cigarette norms. Friend smoking and disagreeing that smokers are marginalised broadly aligned with country-level current smoking rates. Seeing e-cigarette use in public broadly aligned with country-level any e-cigarette use. Generally, no other norms aligned with product prevalence.

## INTRODUCTION

Tobacco smoking is the leading cause of preventable morbidity and mortality worldwide^1,2^. In the European Union (EU), just over a quarter of adults (26%) reported currently smoking tobacco in 2017^3^. However, the nicotine market has changed since the relatively recent introduction of electronic cigarettes (e-cigarettes)^4^, and there has been a rapid increase in their awareness and use in some countries^5-7^. Both combustible tobacco cigarettes and most e-cigarette liquids contain nicotine, the addictive component of smoking. While not entirely absolved from health risks, some reports suggest that e-cigarette use is less harmful than tobacco smoking to both users and people around them, since e-cigarettes do not contain tobacco and do not involve combustion^8-10^. In 2017, 2% of the EU population reported current e-cigarette use^3^.

Social norms towards smoking are often identified as important sources of influence for smoking initiation^11-13^, intention to quit smoking^14-16^, and smoking cessation^14-16^. In the smoking literature, social norms are commonly defined as perceived approval of smoking by friends, family, those important to them, and society (i.e. injunctive norms)^11,12,14,17^, but can also include indicators of perceived visibility, such as self-reported friend smoking and perceptions of how common smoking is (i.e. descriptive norms)^13,18,19^. E-cigarettes, by comparison, are a relatively new product and there is less research on the social norms surrounding them. There have been some debated concerns expressed in the literature^20-22^ and the EU Tobacco Products Directive (TPD) 2014 report^23^ that e-cigarettes might ‘renormalise’ smoking and promote tobacco consumption. Given this, research evaluating social norms towards both e-cigarettes and smoking is of particular importance in the EU.

It is possible that individuals from countries with higher smoking prevalence rates have more pro-smoking social norms. A study among adult smokers in 2002–2003 found that perceived social denormalisation of smoking was lowest in the UK compared with Canada, Australia, and the US^14^; during these years the UK had the highest prevalence of any tobacco smoking of these four countries^24^. Further, a study assessing the 27 countries of the EU found that attitudes towards smoking restrictions were more favourable among those countries with more advanced tobacco control policies and lower smoking prevalence rates^25^. Less is known about country differences in social norms towards e-cigarettes.

**Figure 1:**
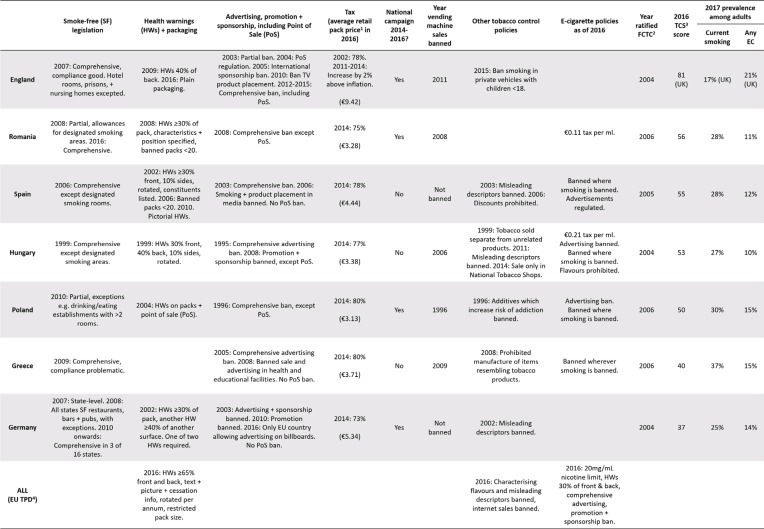
Key tobacco and e-cigarette policies in England, Romania, Spain, Hungary, Poland, Greece, and Germany^3,23,27,28^

Figure 1 shows the prevalence of smoking and e-cigarette use in the seven EU countries of the EUREST-PLUS and International Tobacco Control Policy Evaluation (ITC) Project: Romania, Spain, Hungary, Poland, Greece, Germany, and England. An overview of each country’s tobacco and e-cigarette policy environment is also provided (Figure 1). Of these countries, England had the lowest rates of current smoking (17%) in 2017^3^, accompanied by a strong history of tobacco control policies (Figure 1). Germany, Romania, Spain, Hungary, and Poland have similar rates of current smoking (25–30%), while Greece has the highest current smoking rate (37%) (Figure 1). E-cigarette prevalence rates and policies also differ across these countries (Figure 1); however, *any*, rather than current, e-cigarette use is described, due to low rates of current e-cigarette use and few country differences^3^. England has the highest rates of any e-cigarette use (21%), while Poland, Greece, and Germany (14–15%), and Romania, Spain, and Hungary (10–12%) have similar rates (Figure 1).

The objective of this study was to explore whether social norms towards smoking and e-cigarettes among adult smokers align with smoking and e-cigarette prevalence rates in the seven EU countries of the EUREST-PLUS and ITC Project. It was hypothesised that: 1) social norms will be more pro-smoking among smokers from countries with higher rates of current smoking (i.e. Greece), compared to those from countries with lower rates of current smoking (i.e. England); and 2) social norms will be more pro-ecigarette among smokers from countries with higher rates of any e-cigarette use (i.e. England), compared to those from countries with lower rates of any e-cigarette use (i.e. Hungary, Romania, Spain).

## METHODS

### Pre-registration

The hypotheses, methods and analysis plan were preregistered on the Open Science Framework on 10 May 2018^26^. Hypothesis 2 was changed slightly due to a mistake in the analysis pre-registration, whereby Romania was initially missed.

### Sample

This study is part of the European Regulatory Science on Tobacco: Policy Implementation to Reduce Lung Disease (EUREST-PLUS) Project^29,30^. Data were drawn from Wave 1 of the ITC Six European Country (6E1) Survey (Romania, Spain, Hungary, Poland, Greece, Germany; approximately n=1000 per country) and the England arm of the Wave 1 ITC Four Country Smoking and Vaping (4CV1) Survey (n=3536). These surveys were designed to be nationally representative of current cigarette smokers aged ≥18 years in each country. Survey weights were incorporated to enhance representativeness, and were calculated using information on gender, age, urbanization, and region from national benchmark surveys; further details are provided elsewhere^29-32^.

Data from the ITC 6E1 Survey were collected between 18 June and 12 September 2016. Briefly, data were collected via face-to-face household interviews using tablets (CAPI) and respondents were sampled using a probability approach. Approximately 100 area clusters were sampled in each country, with the aim of obtaining 10 adult smokers per cluster. Within each cluster, household addresses were sampled using a random walk design, and where possible one randomly selected male smoker and one randomly selected female smoker were chosen for interview. Monetary incentives were provided to respondents based on each survey agency’s remuneration structure (Germany, Hungary, Poland €10; Romania €7; Greece €5; Spain €3). Further details are available elsewhere^29-31^.

Data from the ITC 4CV1 England Survey were collected between 7 July and 16 November 2016. Briefly, data were collected online and the majority of respondents were sampled using a non-probability approach. The sample comprised the following cohorts: 1) recontact smokers and quitters living in England who participated in Wave 10 of the earlier 4 Country (4C) Project in the UK; 2) newly recruited current smokers and recent quitters (quit in past 24 months) from a commercial online panel; and 3) newly recruited current e-cigarette users (use at least weekly) from a commercial online panel. In sampling, quotas obtained from national survey data for region crossed with male/female were applied to 2) and 3). Respondents were recruited via random-digit-dialling (RDD) sampling frames, or web-based or address-based panels, or a combination of these frames. Incentives were provided to respondents either in the form of a £16 e-gift card or survey panellist points worth £16–£20. Further details are available elsewhere^32^. Only data from adult current cigarette smokers were used for this study.

### Measures

#### Social norms (outcomes)

The wording of some measures differed between countries. Where wording differed, both measures from the English-translated European Country Surveys^33^, and the England arm of the 4CV1 Survey^34^ are listed separately below. For all social-norms measures, ‘Refused’ and ‘Don’t know’ responses were coded as missing and multiple imputation was used (see Analyses section).

*(i) At least three of five closest friends smoke.* European Survey: ‘Of the five closest friends or acquaintances that you spend time with on a regular basis… How many of them smoke ordinary cigarettes? 0–5’. England Survey: ‘How many friends or acquaintances do you spend time with on a regular basis? 0–5, More than 5’, followed by ‘Of (these 1–5/the 5 closest) friends or acquaintances that you spend time with on a regular basis, how many of them smoke ordinary cigarettes? 0–5’. Responses were dichotomised as less than three (0–2) vs at least three (3–5).

*(ii) People important to you approve of smoking.* ‘What do people who are important to you think about you smoking cigarettes? (a) All or nearly all approve, (b) Most approve, (c) About half approve and half disapprove, (d) Most disapprove, (e) All or nearly all disapprove’. Responses were dichotomised as ‘approve’ (a-b) or ‘not approve’ (c-e).

*(iii) The public approves of smoking. ‘*What do you think the general public’s attitude is towards smoking cigarettes? (a) Strongly approves, (b) Somewhat approves, (c) Neither approves nor disapproves, (d) Somewhat disapproves, (e) Strongly disapproves’. Responses were dichotomised as ‘approve’ (a-b) or ‘not approve’ (c-e).

*(iv) People who smoke are marginalised.* ‘People who smoke are more and more marginalized. (a) Strongly agree, (b) Agree, (c) Neither agree nor disagree, (d) Disagree, (e) Strongly disagree’. Responses were dichotomised as ‘disagree’ (d-e) or ‘not disagree’ (a-c).

*(v) At least one of five closest friends use e-cigarettes.* European Survey: ‘Of the five closest friends or acquaintances that you spend time with on a regular basis… How many of them use e-cigarettes or vaping devices? 0–5’. England Survey: ‘How many friends or acquaintances do you spend time with on a regular basis? 0–5, More than 5’, followed by ‘Of [these 1–5 / the 5 closest] friends or acquaintances that you spend time with on a regular basis, how many of them use e-cigarettes / vaping devices? 0–5’. Responses were dichotomised as none (0) or at least one (1–5), due to the low percentage of respondents who had friends using e-cigarettes.

*(vi) The public approves of e-cigarettes.* European Survey: ‘What do you think the general public’s attitude is towards using e-cigarettes or vaping devices?’ England Survey: ‘What do you think the general public’s attitude is towards vaping/using e-cigarettes? (a) Strongly approves, (b) Somewhat approves, (c) Neither approves nor disapproves, (d) Somewhat disapproves, (e) Strongly disapproves’. Responses were dichotomised as ‘approve’ (a-b) or ‘not approve’ (c-e).

*(vii) Seeing e-cigarette use in public.* European Survey: ‘In the last 30 days, how often have you seen anyone using an e-cigarette or vaping device in public?’ England Survey: ‘In the last 30 days, how often, if at all, have you seen anyone vaping (using e-cigarettes) in public? (a) Every day, (b) Most days, (c) Some days, (d) Rarely, (e) Not at all’. Responses were dichotomised as ‘at least some days’ (a-c), or ‘rarely/not at all’ (d-e).

#### Country

Country was the key correlate: England, Romania, Spain, Hungary, Poland, Greece, Germany.

#### Covariates

*Age:* 18–24, 25–39, 40–54, ≥55.

*Sex:* male, female.

*Household income:* low, moderate, high, not reported.

For England (£) based on annual income: low ≤15000, moderate 15001–30000, high >30000. For the other countries based on monthly income. Germany, Greece and Spain (€): low <1750, moderate 1750–3000, high >3000. For Hungary (Ft): low ≤150000, moderate 150001–250000, high >250000. For Poland (zł): low ≤2000, moderate 2001–4000, high >4000. For Romania (lei): low ≤1000, moderate 1001–2500, high >2500.

*Education:* low, moderate, high.

This variable was defined using the International Standard Classification of Education (ISCED), which was, in turn, categorised into low (pre-primary, primary, lower secondary), moderate (upper secondary, post-secondary non-tertiary, short-cycle tertiary), and high (bachelor or equivalent, master or equivalent, doctoral or equivalent).

*Smoking status:* daily, non-daily.

*E-cigarette status:* current user (use daily, weekly, or occasionally), current non-user.

*Heaviness of Smoking Index (HSI):* 0–6.

The HSI consists of two items: time to first cigarette after waking and number of cigarettes per day^35^. Responses to each item were allocated a score between 0 and 3, and these scores were summed, such that higher values indicate greater heaviness of smoking.

### Analyses

Analyses were conducted using Stata v15^36^. First, the percentages of each social-norms outcome (i–vii) were calculated overall and per country. Second, seven unadjusted and adjusted logistic regression models were used to assess associations between country and each social-norms outcome (i–vii). Adjusted models included all covariates listed above. Stata’s svy command was used for all analyses to account for complex samples design, incorporating survey weights and strata. All frequencies (n) use unweighted, unstratified, ‘raw’ data; all percentages (%) use weighted, stratified data.

#### Missing data

Of the initial 9547 respondents, those who had never heard of e-cigarettes (n=1757) or selected ‘Don’t know’ (n=11) when asked about their e-cigarette status were excluded listwise, leaving 7779 respondents. Missing data were not Missing Completely at Random (MCAR), as country, age, sex, income, education, HSI and e-cigarette use were all associated with missingness. Multiple imputation was therefore used on the remaining sample (n=7779) under the Missing at Random (MAR) assumption for the following ‘Don’t know’ and ‘Refused’ responses: friend smoking (n=721 [9.3%] observations imputed), people important to you approve of smoking (n=415 [5.3%]), public approve of smoking (n=222 [2.9%]), people who smoke are marginalised (n=288 [3.7%]), friend e-cigarette use (n=773 [9.9%]), public approve of e-cigarettes (n=969 [12.5%]), seeing e-cigarette use in public (n=211 [2.7%]), education (n=90 [1.2%]). Multiple imputation was also used on HSI (n=609 [7.8%] observations imputed); this deviated from the pre-registration^26^ due to unanticipated missing values on HSI.

Missing values were imputed using chained equations, and one model was used specifying imputation via logistic regression for all social-norms measures, linear regression for HSI, and ordinal logistic regression for education. Country, age, sex, income, smoking status, and e-cigarette status were included as predictors in the model, and survey weights and strata were incorporated. Forty imputations were used because 31% of respondents had missing data (i.e. responded ‘Don’t know’ or ‘Refused’) on at least one variable included in this study^26^. More respondents from the England sample, who completed the survey online, had missing data on at least one variable (43%) than those from the European samples who completed the survey face-toface (21%). Sensitivity analyses found no differences in the prevalence of any social-norms measure ±1%, in the direction of any odds ratios, or in the significance indicated by p-values at the 0.05 cut-off, when using multiple imputation vs complete case analysis.

## RESULTS

### Sample characteristics

Most respondents were aged 40–54 years, male, had moderate income except Germany (most low or moderate) and England (most high), had moderate education except Germany and Hungary (both majority low education), and were daily smokers but not current e-cigarette users (Table 1).

**Table 1:** Sample characteristics by country, all % (n) except Heaviness of Smoking Index (HSI), which is mean (SD)

	*England (n=3518)*	*Romania (n=679)*	*Spain (n=851)*	*Hungary (n=681)*	*Poland (n=677)*	*Greece (n=737)*	*Germany (n=636)*	*Total (n=7779)*
**Age**								
18–24	16.7 (798)	15.3 (82)	12.9 (106)	9.7 (41)	7.4 (48)	9.6 (51)	9.7 (64)	15.3 (1190)
25–39	32.3 (864)	39.1 (210)	29.6 (266)	35.8 (202)	36.1 (249)	30.8 (209)	24.3 (173)	27.9 (2173)
40–54	26.4 (936)	30.5 (217)	39.7 (287)	33.4 (242)	30.2 (189)	34.5 (285)	37.3 (217)	30.5 (2373)
≥55	24.7 (920)	15.1 (170)	17.8 (192)	21.2 (196)	26.3 (191)	25.0 (192)	28.7 (182)	26.3 (2043)
**Female**	45.9 (1573)	41.0 (272)	43.5 (394)	40.4 (324)	44.6 (366)	47.0 (344)	38.7 (313)	46.1 (3586)
**Income**								
Low	22.4 (771)	15.4 (129)	25.5 (225)	15.4 (117)	13.2 (106)	16.5 (117)	29.8 (191)	21.3 (1656)
Moderate	29.8 (1024)	44.1 (311)	29.7 (241)	27.8 (194)	33.7 (233)	56.8 (398)	29.3 (200)	33.4 (2601)
High	38.4 (1435)	32.9 (182)	6.4 (63)	25.6 (165)	17.7 (112)	10.4 (83)	25.5 (16)	28.4 (2205)
Not reported	9.4 (288)	7.6 (57)	38.4 (322)	31.2 (205)	35.5 (226)	16.4 (139)	15.4 (80)	16.9 (1317)
**Education^a^**								
Low	20.2 (1002)	23.5 (160)	43.3 (342)	61.5 (394)	12.6 (89)	28.3 (201)	50.3 (323)	32.5 (2511)
Moderate	66.2 (1399)	64.4 (436)	48.3 (432)	31.6 (234)	75.2 (492)	49.9 (368)	40.8 (259)	47.3 (3620)
High	13.7 (1051)	12.1 (75)	8.4 (76)	6.9 (51)	12.2 (86)	21.9 (167)	8.9 (52)	20.2 (1558)
**Daily smoker**	83.3 (2866)	96.0 (649)	97.6 (827)	98.9 (673)	96.6 (647)	96.6 (711)	90.9 (578)	89.4 (6951)
**Current EC user**	42.6 (1857)	4.8 (25)	1.3 (10)	3.6 (22)	3.5 (25)	5.4 (41)	9.1 (54)	26.2 (2034)
**HSI^a^**	2.0 (0.0)	2.9 (0.1)	2.3 (0.1)	2.9 (0.1)	2.6 (0.1)	2.9 (0.1)	2.2 (0.1)	2.3 (0.0)

Percentages (%) are weighted and stratified using multiply imputed data. Frequencies (n) are unweighted and unstratified, without multiple imputation.

a Missing data on education (n=90, 1.2%) and HSI (n=609, 7.8%). EC: e-cigarette, HIS: Heaviness of Smoking Index.

### Prevalence of each social-norms measure

Overall, 50% of respondents reported that at least three of their five closest friends smoke, 21% perceived that people important to them approve of smoking, 13% perceived that the public approves of smoking, and 19% disagreed that people who smoke are marginalised (Table 2). Overall, 24% of respondents reported that at least one of their five closest friends uses e-cigarettes, 32% perceive that the public approve of e-cigarettes, and 81% reported seeing e-cigarette use in public at least some days (Table 3). There was substantial difference between countries in smoking (Table 2) and e-cigarette (Table 3) norms; these are examined in further detail below.

**Table 2:** Adjusted associations between each social norm towards smoking measures (i)–(iv) and country (N=7779)

	*(i) At least three of five closest friends smoke*		*(ii) People important to you approve of smoking*		*(iii) The public approves of smoking*		*(iv) Disagree that people who smoke are marginalised*		*Current smoking in 2017^a^*

	*%*	*OR (95% CI)*	*%*	*OR (95% CI)*	*%*	*OR (95% CI)*	*%*	*OR (95% CI)*	*(%)*
**England** (n=3518; ref)									
	19.4	1.00	7.7	1.00	4.9	1.00	9.1	1.00	17
Greece (n=737)									
Unadjusted	83.7	21.38 (16.74–27.32)	18.9	2.77 (2.09–3.69)	16.0	3.73 (2.72–5.12)	50.2	10.11 (8.03–12.73)	37
Adjusted		23.98 (18.25–31.50)		2.84 (2.07–3.91)		5.19 (3.61–7.46)		12.25 (9.39–15.97)	
**Poland** (n=677)									
Unadjusted	69.8	9.59 (7.67–12.00)	25.9	4.17 (3.20–5.43)	19.1	4.63 (3.42–6.25)	16.5	1.99 (1.48–2.67)	30
Adjusted		10.55 (8.14–13.67)		4.62 (3.40–6.27)		6.82 (4.77–9.75)		2.34 (1.69–3.24)	
**Romania** (n=679)									
Unadjusted	82.8	20.01 (15.55–25.75)	28.6	4.79 (3.67–6.25)	21.0	5.19 (3.78–7.12)	38.6	6.32 (4.96–8.05)	28
Adjusted		19.00 (14.48–24.94)		4.44 (3.30–5.98)		5.93 (4.11–8.57)		6.90 (5.24–9.09)	
**Spain** (n=851)									
Unadjusted	73.5	11.53 (9.29–14.33)	13.7	1.90 (1.42–2.54)	5.8	1.20 (0.81–1.76)*	22.9	2.99 (2.36–3.79)	28
Adjusted		11.92 (9.30–15.28)		1.86 (1.34–2.58)		1.69 (1.09–2.60)		3.35 (2.54–4.42)	
**Hungary** (n=681)									
Unadjusted	64.8	7.63 (6.14–9.48)	57.2	15.98 (12.54–20.36)	36.8	11.37 (8.67–14.89)	10.6	1.19 (0.83–1.71)*	27
Adjusted		6.88 (5.37–8.82)		15.12 (11.42–20.03)		15.80 (11.36-21.99)		1.36 (0.94–1.97)*	
**Germany** (n=636)									
Unadjusted	70.9	10.13 (8.08–12.70)	54.9	14.51 (11.43–18.43)	20.9	5.16 (3.83–6.94)	20.5	2.59 (1.99–3.37)	25
Adjusted		11.13 (8.69–14.26)		14.87 (11.35–19.48)		6.59 (4.71–9.21)		2.89 (2.18–3.82)	
**Total** (n=7779)	49.9		21.1		12.9		19.0		

a Data on current smoking rates are from the 2017 Eurobarometer^3^. All data for (i)–(iv) are multiply imputed with survey weights and strata. OR: odds ratio. Adjusted values are adjusted for age, sex, income, education, smoking status, current e-cigarette use and heaviness of smoking index (HSI).

* Data not significant at the p≤0.05 cut-off.

**Table 3:** Adjusted associations between each social norm towards e-cigarette measures (v)–(vii) and country (N=7779)

	*(v) At least one of five closest friends uses e-cigarettes*		*(vi) The public approves of e-cigarettes*		*(vii) Seeing e-cigarette use in public at least some days*		*Any e– cigarette use in 2017^a^*

	*%*	*OR (95% CI)*	*%*	*OR (95% CI)*	*%*	*OR (95% CI)*	*(%)*
**England** (n=3518; ref)							
	28.0	1.00	31.8	1.00	80.5	1.00	21
**Greece** (n=737)							
Unadjusted	27.1	0.96 (0.77–1.19)*	40.1	1.44 (1.18–1.75)	55.1	0.30 (0.24–0.36)	15
Adjusted		1.64 (1.27–2.11)		1.63 (1.31–2.03)		0.39 (0.31–0.49)	
**Poland** (n=677)							
Unadjusted	35.6	1.42 (1.14–1.76)	35.9	1.20 (0.97–1.49)*	44.6	0.20 (0.16–0.24)	15
Adjusted		2.69 (2.06–3.50)		1.46 (1.14–1.85)		0.27 (0.22–0.34)	
**Romania** (n=679)							
Unadjusted	13.5	0.49 (0.30–0.54)	26.1	0.76 (0.59–0.97)	29.5	0.10 (0.08–0.13)	11
Adjusted		0.64 (0.47–0.88)		0.82 (0.63–1.08)*		0.12 (0.09–0.15)	
**Spain** (n=851)							
Unadjusted	5.7	0.15 (0.11–0.21)	23.7	0.67 (0.53–0.84)	12.7	0.04 (0.03–0.05)	12
Adjusted		0.31 (0.22–0.44)		0.81 (0.62–1.04)*		0.05 (0.04–0.07)	
**Hungary** (n=681)							
Unadjusted	23.5	0.79 (0.63–1.00)*	37.0	1.26 (1.01–1.57)	16.9	0.05 (0.04–0.07)	10
Adjusted		1.58 (1.21–2.07)		1.49 (1.17–1.89)		0.06 (0.05–0.09)	
**Germany** (n=636)							
Unadjusted	17.5	0.55 (0.42–0.70)	32.4	1.03 (0.83–1.27)*	28.1	0.09 (0.08–0.12)	14
Adjusted		0.94 (0.72–1.23)*		1.22 (0.97–1.53)*		0.12 (0.10–0.15)	
**Total** (n=7779)	23.6		32.1		53.1		

a Data on any e-cigarette use are from the 2017 Eurobarometer^3^. All data are multiply imputed with survey weights and strata. OR: odds ratio. Adjusted values are adjusted for age, sex, income, education, smoking status, current e-cigarette use and Heaviness of Smoking Index (HSI).

* Data are not significant at the p≤0.05 cut-off.

### Hypothesis 1. Social norms towards smoking will be higher in countries with greater current smoking rates

(i) Reporting that at least three of five closest friends smoke

Both unadjusted and adjusted odds of reporting that at least three of five closest friends smoke were highest in Greece, followed by Romania, Spain, Germany, Poland, Hungary, and lowest in England (Table 2). Odds were 6 to 24 times higher in all countries compared with England, and the results also suggest odds were higher in Greece than all countries except Romania, and in Romania than Poland, Hungary, and Germany (Table 2).

(ii) Perceiving that people important to you approve of smoking

Both unadjusted and adjusted odds of perceiving that people important to you approve of smoking were highest in Hungary, followed by Germany, Romania/Poland, Greece, Spain, and lowest in England (Table 2). Odds were 1.8 to 16 times higher in all countries compared with England, and the results also suggest odds were higher in Hungary and Germany than all other countries, and in Poland and Romania than Spain (Table 2).

(iii) Perceiving that the public approves of smoking

Unadjusted odds of perceiving that the public approves of smoking were highest in Hungary, followed by Romania, Germany, Poland, Greece, Spain, and England, while adjusted odds were highest in Hungary, followed by Poland, Germany, Romania, Greece, Spain, and lowest in England (Table 2). The results suggest odds were lower in England and Spain compared with all other countries, higher in Hungary than all countries, and adjusted odds were also lower in England than Spain (Table 2).

(iv) Disagreeing that people who smoke are marginalised

Both unadjusted and adjusted odds of disagreeing that people who smoke are marginalised were highest in Greece, followed by Romania, Spain, Germany, Poland, Hungary, and lowest in England (Table 2). The results suggest odds were lower in England and Hungary than all other countries, higher in Greece and Romania than all other countries, and adjusted odds were also higher in Greece than Romania (Table 2).

### Hypothesis 2. Social norms towards e-cigarettes will be higher in countries with greater rates of any e-cigarette use

(v) Reporting that at least one of five closest friends uses e-cigarettes

Unadjusted odds of reporting that at least one of five closest friends uses e-cigarettes were highest in Poland, followed by England, Greece, Hungary, Germany, Romania, and lowest in Spain (Table 3). Adjusted odds were highest in Poland, followed by Greece, Hungary, England, Germany, Romania, and lowest in Spain (Table 3). The results suggest odds were generally higher in Poland compared with all countries except Greece, higher in Greece than Romania and Germany, higher in England and Hungary than Romania, and lower in Spain than all countries (Table 3).

(vi) Perceiving that the public approves of e-cigarettes

Unadjusted and adjusted odds of perceiving that the public approves of e-cigarettes were highest in Greece, followed by Hungary, Poland, Germany, England, Romania, and lowest in Spain (Table 3). The results suggest odds were generally higher in Greece, Poland and Hungary than England, Romania, and Spain (Table 3).

(vii) Report seeing e-cigarette use in public at least some days

Unadjusted and adjusted odds of reporting seeing e-cigarette use in public at least some days was highest in England, followed by Greece, Poland, Romania, Germany, Hungary, and lowest in Spain (Table 3). Odds were 2.6 to 25 times higher in England compared with all countries, and the results also suggest higher odds in Greece and Poland than all other countries except England, and lower in Spain and Hungary than all other countries (Table 3).

## DISCUSSION

Partially consistent with Hypothesis 1, smokers from countries with higher rates of current smoking generally had more pro-smoking social norms on two of four measures: reporting that at least three of their five closest friends smoke, and disagreeing that smokers are marginalised. Except England, generally perceived approval of smoking by those important to you and society did not align with country-level rates of current smoking. Somewhat consistent with Hypothesis 2, smokers from countries with higher rates of any e-cigarette use had more pro-e-cigarette social norms on one of three measures: seeing e-cigarette use in public at least some days. Generally, reporting that at least one of five closest friends uses e-cigarettes and perceiving that the public approves of e-cigarettes did not align with country-level rates of any e-cigarette use. Smokers from England had the least pro-smoking social norms across all four measures and countries, while those from Spain had the least pro-e-cigarette social norms across all three measures and countries.

The finding that England had the least pro-smoking norms across all four measures is unsurprising, given England’s substantially lower smoking rate and long history of strong tobacco control policies compared with the other six EU countries in this study (Figure 1). However, England did not have the most pro-ecigarette social norms on two of three measures, despite its markedly higher country-level rates of any e-cigarette use compared with the other six countries (Figure 1) and some promotion of e-cigarettes as smoking cessation aids by UK public health bodies such as the NHS and Cancer Research UK.

The finding that smokers from Spain had the least pro-e-cigarette social norms across all three measures also warrants further exploration, given that prevalence of any e-cigarette use in Spain was not markedly different from any other country’s except England. Public health authorities in Spain have generally applied precautionary principles towards e-cigarettes, such as banning their use in most public places and workplaces in 2014 (Figure 1). There has also been a delay in the general marketing of e-cigarettes in Spain compared with other countries. It should also be noted that Spanish smokers’ low perceived approval of smoking, from those important to them (14%) and the public (6%), is not consistent with the higher smoking prevalence in Spain.

Averaged across all seven countries, perceived public approval of e-cigarettes (32%) was over twice that of perceived public approval of smoking (13%). Moreover, perceived public approval of e-cigarettes was also higher than that of smoking within all countries. This is consistent with reports suggesting e-cigarettes are less harmful to both users and people around them relative to combustible cigarettes^8-10^. It is not possible to compare the other social norms towards smoking with those social norms towards e-cigarettes due to different types of social norms being assessed.

This study is among the first in the EU to assess a variety of both descriptive, more ‘visible’ measures of adult smokers’ social norms towards smoking and e-cigarettes, such as perceived friend use and seeing e-cigarette use in public, in addition to injunctive norms such as perceived approval. The results suggest that the injunctive norms measured here do not align with country-level rates of product use, nor do they generally correspond with the descriptive norms. Given literature highlighting the importance of measures of both the perceived visibility of smoking and perceived approval of smoking^18^, future research should aim to consider both normative domains.

There are several potential explanations as to why smokers’ perceived approval of smoking by those important to them and that the public did not align with country-level current smoking rates as hypothesised. First, Hypothesis 1 was based on 2017 current smoking prevalence, which fails to consider each country’s history of smoking prevalence, and current and previous tobacco control policies. These likely play important roles. Second, the sample was limited to current smokers, who are more likely to be of lower socioeconomic status and lacking the motivation and resources to quit^37^. Such individuals may hold more entrenched or polarised social norms; indeed, current smokers have been found to hold more pro-smoking norms across many self-report measures compared to non-smokers and exsmokers^19,38^. Therefore, perceived approval of smoking *among current smokers* may be amplified in countries where they are in the minority, although this was not the case in England. Studies assessing smoking, and e-cigarette, social norms among non-smokers and ex-smokers may aid interpretation of these findings.

The finding that social norms towards e-cigarettes, generally, did not align with country-level rates of any e-cigarette use as hypothesised could also be attributed to the sample containing current smokers only. In the EU, e-cigarettes are often used as an aid to smoking cessation^3,39^, and some smokers may be encouraged to switch from smoking to e-cigarette use due to the health benefits of switching over continued smoking^7^. Given this, it makes some sense that smokers from countries with historically higher rates of current smoking, such as Greece and Poland, would have greater adjusted odds of friend e-cigarette use and perceived public approval of e-cigarettes. However, this explanation is anecdotal and requires further research. Further, any e-cigarette use is a relatively weak measure of prevalence, yet options for a more refined comparator for Hypothesis 2 were limited, since prevalence of current e-cigarette use was low and similar for each country (<1–5%)^3^. Current and previous e-cigarette policies were also not considered. Other potential explanations pertaining to the unanticipated results for both smoking and e-cigarette social norms include cross-country differences in culture, freedom of speech, liberty and social connectedness, which likely all play a role in the development of social norms^40^.

## Limitations and strengths

This study is not without limitations. First, the results have limited generalizability since the sample contained only current smokers, who generally hold more pro-smoking norms across many self-report measures^19,38^, and have been found to perceive greater public approval of e-cigarettes^19^, compared to nonsmokers and ex-smokers. Second, the seven EU countries included in this study have all been working to reduce tobacco smoking through strengthening policies over the past decade, and all have some tobacco and e-cigarette policies harmonised under EU legislation. Inclusion of countries at an earlier stage of the tobacco epidemic or with considerably less restrictive tobacco control policies may have aided the interpretation of findings. Third, the English sample differed on the wording of some survey items, used online rather than face-to-face methodology, were offered greater monetary incentives, and had more missing data than the other EU country samples. This weakens comparisons made between England and the other countries. Fourth, smokers’ understanding of these social-norms measures may differ across the different languages used, and may be subject to cultural biases^41^. Despite these limitations, this study is the first of its kind to compare social norms towards smoking and e-cigarettes in different EU countries, and uses large, nationally representative samples.

## CONCLUSIONS

Among current smokers from seven EU countries, those from England had the least pro-smoking social norms, while those from Spain had the least pro-e-cigarette social norms. Reporting that at least three of five closest friends smoke and disagreeing that smokers are marginalised broadly aligned with country-level rates of current smoking, being lowest in England and highest in Greece. Seeing e-cigarette use in public broadly aligned with country-level any e-cigarette use, being lowest in Hungary, Romania and Spain, and highest in England. No other social norms were consistent with smoking and e-cigarette prevalence rates as hypothesised.
